# Neurophysiological Basis of Deep Brain Stimulation and Botulinum Neurotoxin Injection for Treating Oromandibular Dystonia

**DOI:** 10.3390/toxins14110751

**Published:** 2022-11-02

**Authors:** Hitoshi Maezawa, Masayuki Hirata, Kazuya Yoshida

**Affiliations:** 1 Faculty of Rehabilitation, Kansai Medical University, Uyama-higashi 18-89, Hirakata 573-1136, Japan; 2 Department of Neurological Diagnosis and Restoration, Graduate School of Medicine, Osaka University, Yamadaoka 2-2, Suita 565-0871, Japan; 3 Department of Oral and Maxillofacial Surgery, National Hospital Organization, Kyoto Medical Center, 1-1 Mukaihata-cho, Fukakusa, Fushimi-ku, Kyoto 612-8555, Japan

**Keywords:** oromandibular dystonia, botulinum neurotoxin, stomatognathic function, deep brain stimulation, sensorimotor function, magnetoencephalography, electroencephalography, motor function, globus pallidus

## Abstract

Oromandibular dystonia (OMD) induces severe motor impairments, such as masticatory disturbances, dysphagia, and dysarthria, resulting in a serious decline in quality of life. Non-invasive brain-imaging techniques such as electroencephalography (EEG) and magnetoencephalography (MEG) are powerful approaches that can elucidate human cortical activity with high temporal resolution. Previous studies with EEG and MEG have revealed that movements in the stomatognathic system are regulated by the bilateral central cortex. Recently, in addition to the standard therapy of botulinum neurotoxin (BoNT) injection into the affected muscles, bilateral deep brain stimulation (DBS) has been applied for the treatment of OMD. However, some patients’ OMD symptoms do not improve sufficiently after DBS, and they require additional BoNT therapy. In this review, we provide an overview of the unique central spatiotemporal processing mechanisms in these regions in the bilateral cortex using EEG and MEG, as they relate to the sensorimotor functions of the stomatognathic system. Increased knowledge regarding the neurophysiological underpinnings of the stomatognathic system will improve our understanding of OMD and other movement disorders, as well as aid the development of potential novel approaches such as combination treatment with BoNT injection and DBS or non-invasive cortical current stimulation therapies.

## 1. Introduction

The stomatognathic system is an anatomic and functional unit comprising the mandible, maxilla, dental arches, teeth, temporomandibular joint, masticatory muscles, surrounding nerves and vessels, and salivary glands. The stomatognathic system plays important roles in a variety of critical motor functions, including mastication, swallowing, speech production, and respiration. Focal dystonia in the stomatognathic system induces severe motor dysfunction that negatively affects quality of life via factors such as masticatory disturbances, limited mouth opening, dysphagia, and dysarthria caused by involuntary movements in the stomatognathic system [1,2,3].

Dystonia is characterized by sustained or intermittent muscle contractions that produce involuntary, unwanted, and often repetitive movements, postural changes, or both [4]. Focal dystonia includes blepharospasm (eyelids), cervical dystonia (neck), writer’s cramp, musician’s cramp (hand and arm), and oromandibular dystonia (OMD) [5]. OMD is a focal type of dystonia that involves the masticatory, lower facial, lingual, or orbicularis oris muscles [1,2,3,6]. Clinically, OMD presents as dystonia during jaw opening or closing, jaw deviation, jaw protrusion, lingual dystonia, lip dystonia, or a combination of these abnormal movements [7].

When OMD occurs in conjunction with blepharospasm, it is usually referred to as Meige syndrome. Meige syndrome, which was first described by Henri Meige in 1910, is cranial dystonia characterized by the combination of upper and lower cranial involvement and including blepharospasm and OMD [8]. In Meige’s syndrome, there is progressive worsening of blepharospasm with the spread of symptoms to involve the oromandibular, cervical, and limb muscles; however, the symptoms spread earliest and greatest in the oromandibular muscles [9,10,11].

A recent multicenter study conducted among Institutions across seven countries found that OMD had a prevalence of 8.7% among all types of focal dystonia, making it one of the less prevalent subtypes of this neurological disorder [12]. The overall prevalence of primary dystonia was calculated as 16.4 per 100,000 cases in a meta-analysis [13], and that of OMD was estimated at 6.8 per 100,000 cases [14]. Another study reported an estimated OMD prevalence rate of 0.33 per 100,000 cases [15]. However, most of the studies evaluated relatively few patients with OMD. A recent epidemiologic study of 144 patients with OMD reported an estimated prevalence of 9.8 per 100,000 cases, suggesting that OMD is as common as cervical dystonia or blepharospasm [16].

Botulinum neurotoxin (BoNT) injection represents a standard therapy for focal dystonia, including cases of OMD [3,17]. BoNT is injected into the target muscles exhibiting disordered movements where it blocks acetylcholine release at the neuromuscular junction (NMJ). Proper muscle identification, dose selection, and management of patient expectations are required to ensure that BoNT injection is both safe and effective in patients with OMD [3], since stomatognathic functions are performed by various bilateral muscles in the stomatognathic system that differ in size and tension. Moreover, since each muscle of the stomatognathic system is controlled by the bilateral cortex through the corticobulbar tract and certain cranial nerves (CNs (CN V, VII, X, XII)), identifying the neurophysiological and neuroanatomical basis of sensorimotor functions in the stomatognathic system remains essential.

**Figure 1:**
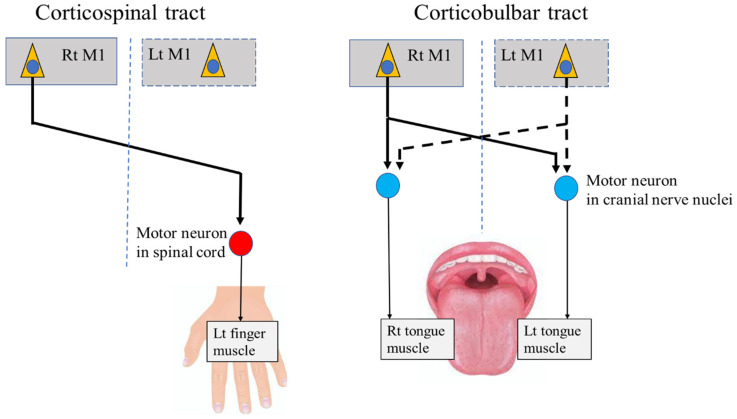
Corticospinal tract and corticobulbar tract. M1: primary motor cortex, Rt: right, Lt: left.

In addition to BoNT injection, deep brain stimulation (DBS) represents an optional therapy for focal dystonia in the limbs [18]. The main target of conventional DBS is the internal segment of the unilateral globus pallidus (GPi). DBS of the unilateral GPi improves symptoms of disordered movement on the contralateral side, based on the principle that unilateral movements in the limbs are regulated by the contralateral sides of the central regions via the corticospinal tract (Figure 1). However, the application of DBS for OMD has been limited, and evidence regarding its clinical utility in these patients is still considered preliminary.

Several case reports from the past two decades have described the use of DBS of the bilateral Gpi for the treatment of lingual dystonia [19,20,21,22]. This bilateral approach is based on the neurophysiological principle that tongue movements are regulated by the bilateral central regions through the corticobulbar tract, which differs from the contralateral regulation of the limbs (Figure 1). The uncertain effects of DBS on OMD may, in part, be explained by the unique characteristics of the bilateral central regulation of sensorimotor functions in the stomatognathic system. To establish effective treatments targeting the central regions, including DBS, further research is required to elucidate the neurophysiological basis and central mechanisms related to stomatognathic functions.

Recent advances in non-invasive electromagnetic physiological techniques, such as electroencephalography (EEG) and magnetoencephalography (MEG), have enabled the investigation of cortical mechanisms in humans with high temporal resolution. In particular, MEG is advantageous owing to its high spatial resolution given that the magnetic permeability of biological tissues is nearly identical to that of empty space; thus, the magnetic field is not distorted by the scalp or skull [23]. 

In this review, we present an overview of the unique central mechanisms of bilateral spatiotemporal processing associated with the sensorimotor functions of the stomatognathic system. These mechanisms have been demonstrated in MEG/EEG studies using multiple parameters for analysis, including somatosensory evoked fields/potentials (SEFs/SEPs), movement-related cortical fields/potentials (MRCFs/MRCPs), and cortico-muscular/cortico-kinematic coherence (CMC/CKC). We also discuss the pathophysiological characteristics of typical cases of hand dystonia, such as writer’s cramp and OMD, as demonstrated using EEG and MEG. Finally, we identify current problems and future directions in OMD treatment. 

## 2. Neural Pathways from the Primary Motor Cortex (M1) to Stomatognathic Systems

In the human primary motor cortex (M1), the cortical representations of various body parts are organized in the form of the “classical homunculus” [24]. In general, the homunculus resembles an upside-down map of each body part, in which the stomatognathic systems are closer to the lateral sulcus than the upper and lower extremities. The area of the M1 that represents the stomatognathic system is widely distributed relative to its actual size in the body [24], suggesting a rich innervation of these areas. 

Anatomically, each muscle of the stomatognathic system receives innervation from the bilateral M1 through the corticobulbar tract, which terminates in motor neurons within the CN nuclei (Figure 1). In contrast, the muscles of the upper and lower limbs are predominantly innervated by the contralateral cortex through the corticospinal tract, which terminates in motor neurons within the spinal cord (Figure 1). Indeed, a previous study in patients with epilepsy showed that direct unilateral cortical stimulation of the M1 areas for the oral and upper limb muscles induced bilateral oral movements but unilateral limb movements [24].

The stomatognathic system is composed of various muscles, including the tongue muscles, orbicularis oris, and masticatory muscles responsible for jaw closing (masseter, temporalis, and medial pterygoid) and opening (inferior head of lateral pterygoid, anterior belly of digastric, geniohyoid, and mylohyoid). All the muscles of the tongue are innervated by the hypoglossal nerve (CN XII), except for the palatoglossal muscles, which are innervated by the vagus nerve (CN X). Masticatory muscles are innervated by the trigeminal nerve (CN V), except for the posterior bellies of the digastric muscles, which are innervated by the facial nerve (CN VII). The orbicularis oris muscle is innervated by the facial nerve (CN VII).

Moreover, bilateral innervation contributes to somatosensory sensations in the stomatognathic system. Somatosensory information from the tongue is transmitted by the mandibular branch of the trigeminal nerve (CN V) (anterior two-thirds of the tongue), glossopharyngeal nerve (CN IX) (posterior one-third of the tongue), and vagus nerve (posterior part of the tongue root) (CN X). Somatosensory information from the soft palate, teeth, gingiva, face, and lips is transmitted by the mandibular or maxillary branches of the trigeminal nerve (CN V). Proprioceptive sensations from the tongue muscle are transmitted by the hypoglossal nerve (CN XII) and its rich innervation of the related muscle spindles. Proprioceptive sensations from the masticatory muscles are mainly transmitted by the trigeminal nerve (CN V) for jaw-closing muscles, as the jaw-closing muscles have many more muscle spindles than the jaw-opening muscles [25]. 

## 3. Bilateral Brain Activation Related to Stomatognathic Functions

A previous study involving non-invasive, whole-head MEG successfully identified the classical homunculus in the primary somatosensory cortex following tactile stimulation of multiple points on the human body [26]. Cortical representations of oromandibular areas are widely distributed in the primary sensorimotor cortex relative to their actual size in the human body [26]. In this section, we describe in detail the unique central mechanisms of bilateral spatiotemporal information processing involved in the sensorimotor functions of the stomatognathic system, as demonstrated using MEG/EEG.

### 3.1. Movement-Related Cortical Fields and Potentials

In early studies, researchers observed slowly increasing cortical fields over the contralateral hemisphere before the onset of voluntary unilateral movements of a finger, which they termed MRCFs [27,28]. Cheyne et al. [29] first reported MRCFs associated with repetitive tongue protrusion in a patient using a seven-channel MEG system to record from the left hemisphere. Nakasato et al. [30] demonstrated whole-head MRCFs for tongue protrusion in five healthy volunteers, using a trigger signal that was detected when the tip of the tongue reached the anterior region of the palate. They reported that the peak magnitude of the MRCFs was derived from the areas of the bilateral M1 region representing the tongue. A recent MEG study investigated the characteristics of bilateral MRCFs before and after voluntary self-paced tongue movements, using surface electromyography (EMG) from the tongue [31]. As reported in previous MRCF studies of finger movement [27,28,32], three components were detected in response to tongue movement: the readiness field (RF), motor field (MF), and movement-evoked field (MEF). Slowly increasing RF components were observed bilaterally before the onset of movement, peaking in MFs around the onset of movement. The MF component appeared after the pre-movement RF component and originated from part of the primary motor area (M1) representing the tongue. The MEF component, which appears after movement onset and originates from the bilateral primary somatosensory area (S1), may reflect proprioceptive feedback from the tongue during voluntary tongue movement. These results suggest that the bilateral M1 and S1 are involved in the preparation and execution of tongue movements [31].

MRCPs are slowly increasing cortical shifts in negative potentials that occur prior to voluntary movements, as demonstrated using EEG [33]. In an EEG study of MRCPs related to jaw movement, a previous study [33] reported a slowly increasing negative potential originating 1.5–2 s before EMG onset, known as the Bereitschaftspotential (BP) [34,35]. The BP, also called the readiness potential, is a measure of brain activity in the motor cortex and supplementary motor area leading up to voluntary muscle movement. The maximum BP occurred over the vertex region, and the negative slope (NS’) occurred approximately 300–700 ms before EMG onset. The authors reported that BP/NS’ amplitudes at the onset of movement differed significantly among open, closed, and lateral movements. The MRCPs at mouth opening and closing were symmetrically distributed, whereas those at lateral movements were predominant over the hemisphere ipsilateral to the direction of movement. 

In an MEG study of jaw movement in five healthy volunteers [36], RFs were observed bilaterally, starting around 860 and 600 ms prior to the onset of masseter and digastric EMGs, respectively, and gradually increasing in magnitude until peaking within 100 ms before EMG onset. In all participants, the equivalent current dipoles generating the RFs accompanying jaw-opening movements were located in the bilateral M1 areas representing the jaw. These results suggest that MRCF/MRCP recordings provide a means for exploring the spatiotemporal characteristics of the sensorimotor cortex before and after voluntary movements in the stomatognathic system.

### 3.2. Cortico-Muscular Coherence and Cortico-Kinematic Coherence during Voluntary Movements

**Figure 2:**
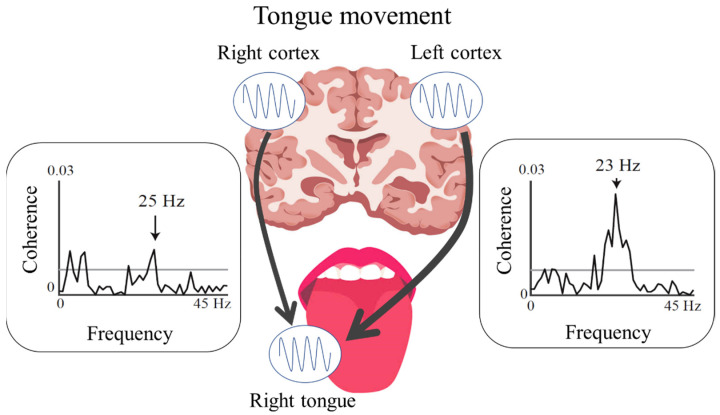
Bilateral functional connections between the cortex and tongue identified using cortico-muscular coherence analysis. The figure shows contralateral (left hemispheric) dominance of the functional connection between the cortex and right side of the tongue.

CMC analyses can aid in evaluating oscillatory functional connections between the motor cortex and corresponding peripheral muscles during sustained muscle contraction [37,38]. The CMC in the β-frequency band (β-CMC) during the sustained unilateral movements of a finger mainly represents the descending motor commands from the M1 to the contralateral finger muscle through corticospinal pathways [38]. In our previous MEG study [39], the CMC of the tongue was detected at two different frequency bands (the β-band and a low-frequency band at 2–10 Hz) over both hemispheres for each side of the tongue during isometric tongue protrusion [39,40]. The β-CMC for the tongue mainly reflects the descending motor commands from each side of the M1 to both sides of the tongue, with contralateral dominance through hypoglossal motoneuron pools (Figure 2). Moreover, the somatotopic organization of the tongue and finger regions in the M1 allows for differentiation of the cortical source locations of the β-CMC for the tongue and finger [39]. In contrast to β-CMC, CMC in the low-frequency band (low-CMC) reflects oscillatory coupling related to proprioceptive feedback from the tongue muscles to the bilateral S1 [40]. This pattern of central regulation with oscillatory activity in two frequency bands is a unique characteristic of stomatognathic functions, since a stable low-CMC is not detected during finger movements. 

CKC methods are used to quantify the coupling between MEG signals and finger kinematics, which are measured using an accelerometer during repetitive, rhythmic, or voluntary finger movements [41]. A recent MEG study has demonstrated that CKC data mainly reflect proprioceptive sensory feedback from the peripheral muscles to S1 [42]. However, applying conventional CKC to stomatognathic systems is difficult, as magnetic accelerometer devices can easily produce excessive magnetic artefacts due to the short distance between the brain and stomatognathic systems. To overcome this limitation, a recent MEG study successfully utilized a deep-learning-assisted motion capture system, in which the CKC was detected during rhythmic tongue movements at the frequency peaks of movement or its harmonics, based on signals in the bilateral primary sensorimotor cortex representing the tongue regions [43]. This combination of CKC and deep learning-assisted motion capture has the advantage of being noise-, movement-, and risk-free, particularly in the oral regions, because recording devices are not placed in the orofacial region. As the tongue muscles and those responsible for closing the jaw are rich in muscle spindles [25], the CKC approach may help to reveal the central proprioceptive mechanisms of the stomatognathic system and the pathophysiology of OMD involving impairments in proprioceptive pathways.

### 3.3. Somatosensory-Evoked Fields and Potentials

Since the first reports of SEFs induced by trigeminal nerve stimulation in the early 1980s [44] and 1990s [45], many MEG studies have sought to assess SEF characteristics in the tongue [45,46,47,48], lips [49,50], palate [51,52], teeth [44], gingiva [49], and lower face [53].

Following tongue stimulation, the initial SEF components occur over the bilateral hemispheres at 19 ms with electrical stimulation [47] and 15 ms with tactile stimulation [48], with an anterior current orientation. These timings are consistent with a SEP latency of 16 ms following electrical stimulation of the lip via chronically implanted subdural electrodes over the lower perirolandic area in patients undergoing epilepsy surgery [54]. However, the initial component of trigeminal SEF/SEP is not detected stably due to the low amplitude of this component and is easily masked by excessive stimulation artefacts. Thus, in clinical settings, a prominent component with a large amplitude at a middle latency ranging from 25 ms to 80 ms, with a posterior current orientation, is often adopted [45,46,49,52,53,55]. Indeed, Previous studies reported that the middle-latency component of the trigeminal SEF can be used to evaluate sensory disturbances of the tongue and lip caused by injury to the trigeminal nerve during dental surgery [50,56,57]. Source localization studies involving MEG with high spatial resolution have revealed that the initial and middle-latency components of SEFs stimulated in the stomatognathic system are derived from the bilateral S1, specifically, the posterior bank of the central sulcus [46,47,48,49,50]. This neurophysiological finding is consistent with the anatomical principle that the unilateral lingual nerve innervating the anterior part of the tongue projects to both sides of area 3b in S1 via the trigeminothalamic tract, with contralateral dominance.

## 4. Oromandibular Dystonia

### 4.1. Pathophysiology of OMD

Although the pathogenesis of primary dystonia is still a matter of debate, several probable explanations have been proposed, including basal ganglia dysfunction, hyperexcitability of motor neurons, loss of inhibition, aberrant dopamine signaling, monoaminergic dysfunction, abnormal plasticity, and abnormal sensory function [58,59,60,61]. Most previous EEG studies of focal dystonia in humans have focused on cortical sensorimotor dysfunction, especially of the primary sensorimotor cortex and premotor areas, in cases affecting the hand (e.g., writer’s cramp) [59].

MRCPs associated with hand movements consisted of at least two slow negative shifts. The early component is the BP, which is maximal at the vertex and symmetrically distributed over the scalp, whereas the latter component is called NS’, which is largest in the central area contralateral to the hand movement [62]. The BP represents the activation of supplementary motor areas (SMAs), while NS’ reflects activities in both the SMA and M1 [63,64,65]. However, the precise anatomical representations of the BP and NS’ remain controversial.

Several EEG studies have reported abnormalities in MRCPs in patients with focal dystonia [66,67,68]. Reductions in both NS’ and BP gradients have been observed in patients with symptomatic dystonia caused by lesions in the basal ganglia and anterior thalamus. Furthermore, reductions in NS’ amplitude over the contralateral central region have been observed in patients with writer’s cramp [66,67]. These results suggest impaired activation of the sensorimotor cortex contralateral to the affected hand immediately before a voluntary movement. One study also reported abnormalities in the cortical preparatory processes for voluntary muscle relaxation or motor inhibition in patients with focal hand dystonia [68]. The BP associated with voluntary muscle relaxation was reduced over the central region of the affected hemisphere in patients with focal hand dystonia, suggesting impaired activation of the inhibitory motor systems in the contralateral cortex in these patients [68]. Moreover, a functional magnetic resonance imaging (fMRI) study reported smaller activation volumes in the SMA proper and primary sensorimotor cortex during voluntary muscle contraction and relaxation of the affected (right) hand in patients with writer’s cramp than in healthy volunteers [69]. Disturbances in sensorimotor integration have also been documented as a task-specific decrease in the amplitude of contingent negative variation [70,71], abnormal pre-movement gating of somatosensory input [72], and abnormal CMC with MEG [73]. Other studies using paired-pulse transcranial magnetic stimulation have reported impairments in short-interval intracortical inhibition [74] and surround inhibition [75]. Somatotopic disorganization of the fingers has also been observed in the S1 contralateral to the affected hand [76]. These findings collectively demonstrate that focal dystonia of the hand involves impaired cortical inhibition in M1, abnormal sensorimotor integration, and disorganization of S1 [69,70].

A few EEG studies have also investigated the pathological characteristics of OMD. A study compared MRCPs associated with mandibular movements in six patients with OMD and eight healthy controls [35]. In the patient group, MRCP amplitudes over the central and parietal areas for mouth opening and lateral movements were significantly reduced compared to those observed in the control group. Moreover, in controls, the MRCPs at mouth opening and closing were symmetrically distributed, whereas those at lateral movements were predominant over the hemisphere ipsilateral to the direction of movement. This laterality was lost in the patient group. These results suggest that OMD is associated with bilateral impairments in cortical preparatory processing during jaw movement. A few studies have also investigated OMD pathology using fMRI and positron emission tomography. One fMRI study reported reduced activation of the primary sensorimotor cortex and premotor/sensory association cortices during vocalization in patients with laryngeal dystonia [77]. Analogous to the motor dysfunction observed in patients with dystonia, reduced primary somatosensory activity has previously been demonstrated [78,79], supporting the conceptualization of dystonia as a partial sensory disorder [80,81].

### 4.2. Treatment Options for OMD

BoNT injection represents the standard therapy for patients with focal dystonia such as blepharospasm, cervical dystonia, and OMD [82,83,84]. When the target muscles, doses, and patient expectations are appropriately managed, BoNT injection is a safe and effective approach for the treatment of OMD [3]. Some studies reported the effectiveness of ultrasound-guided injections in facial muscles to avoid injuring the neural vascular structures during botulinum toxin injection [85,86].

BoNT is a microbial protein that exists in seven serotypes, designated A through G. Its ability to block acetylcholine release at the NMJ accounts for its therapeutic effects in various movement disorders associated with increased muscle tone or muscle overactivity. BoNT wields these effects by entering nerve endings at the NMJ and cleaving soluble N-ethylmaleimide-sensitive factor-attachment protein receptors, thereby preventing the vesicular release of acetylcholine from the synaptic terminal and producing the effects of muscle relaxation and flaccid paralysis [87,88].

In addition to the effect on the peripheral nervous system, BoNT may also indirectly influence the functional organization of the central nervous system associated with altered peripheral inputs [89,90]. Moreover, the central effect of BoNT injection is not limited to the cortical and subcortical regions of the treated muscles but extends beyond the neural circuits for the control of the affected body parts [89,91,92]. Further studies with non-invasive brain-imaging techniques, including MEG and EEG, may help to reveal the bilateral cortical areas affected by therapy with BoNT injection in patients with OMD.

Other treatments for focal dystonia include invasive approaches, such as DBS, targeting central regions [93,94,95]. When symptoms cannot be adequately managed through medication and rehabilitation, DBS is typically employed as a surgical intervention to treat movement disorders. The main DBS target for dystonia treatment is the internal segment of the GPi [96], which has proven efficacious in the treatment of generalized, segmental, and cervical dystonia [97,98,99]. Although evidence concerning the efficacy of DBS for other dystonia subtypes remains scarce, DBS targeting the GPi has demonstrated continued efficacy in patients with Meige syndrome [94,100,101,102]. A recent meta-analysis showed that DBS may be useful for treating refractory Meige syndrome [103]. However, other studies have demonstrated that in some patients with craniocervical and craniofacial segmental dystonia, DBS is ineffective for improving stomatognathic functions such as speech and swallowing but is effective for improving blepharospasm [95,104,105]. A recent study of DBS in 18 patients with dystonia related to *KMT2B* mutations reported that the greatest improvements in motor function were observed in patients with trunk and cervical dystonia, with less clinical impact observed in patients with laryngeal dystonia [106].

Currently, only three published case reports have discussed bilateral GPi-DBS for OMD, all of which were categorized as lingual dystonia. Chung et al. [20] and Asahi et al. [22] reported symptomatic improvements in lingual dystonia following bilateral GPi-DBS in two cases and one case, respectively. In patients with OMD, the unique neuroanatomical characteristics of bilateral innervation in each side of the stomatognathic system may complicate the application of conventional DBS compared to that in patients with focal dystonia of the limbs, as previous studies have reported that sufficient effects are sometimes difficult to obtain with interventions targeting the unilateral hemisphere. As contralateral dominance is involved in the control of voluntary movement in the stomatognathic system, it may be beneficial for future research to explore the most effective stimulation parameters for bilateral DBS targeting the GPi in patients with OMD. However, some patients’ OMD symptoms do not improve sufficiently even after DBS, and they require additional BoNT therapy. A highly individualized injection technique has been developed for lingual dystonia according to the direction of deviation [107]. An improved understanding of the central and peripheral mechanisms related to stomatognathic functions may promote the development of new treatments for OMD, such as the combination of BoNT injection into the affected muscles as peripheral therapy and DBS as central therapy.

Several authors have also discussed the use of unique oral appliances (e.g., oral splints) for OMD treatment [108,109,110,111,112,113]. Previous research has shown that performing certain sensory tricks (e.g., pressing the teeth or lips with the fingers; placing cigarettes, chewing gum, or other objects in the mouth; singing; and humming) may benefit a third of patients with OMD [109,114,115]. Oral appliances may be particularly useful in cases where they successfully mimic the patient’s sensory tricks [109,114], as treatment responses in such patients may be linked to sensory tricks perceived by the brain to be beneficial. Although the precise mechanism underlying this phenomenon remains unknown, the beneficial effect of splint use on OMD may be related to the modulation of hyperactive dystonic networks by altered proprioceptive feedback and antagonist activation [114]. MEG recordings with CKC and low-CMC analyses are useful for evaluating proprioceptive feedback from muscles to the cortex during voluntary movements in the stomatognathic system (see Section 3.2). These approaches may aid in revealing the effects of oral appliance therapy on abnormal cortical activity related to proprioceptive feedback in patients with OMD.

## 5. Future Directions

BoNT injection is the standard peripheral therapy for patients with OMD. In addition, central therapies such as bilateral DBS can be applied, providing substantial relief in patients with disordered movements. However, the introduction of stimulating electrodes deep in the brain carries significant risks, including the risk of hemorrhage [116]. Beyond DBS, recent studies have highlighted the potential of non-invasive cortical current stimulation therapies such as transcranial direct current stimulation (tDCS) [117,118] and transcranial alternating-current stimulation (tACS) [119] for patients with limb movement disorders, although their effectiveness and mechanisms of action are still unclear [120,121,122]. For example, a previous study successfully reported the non-invasive application of tACS over the M1 [119], which induced phase cancellation of the resting tremor rhythm in the upper limbs of healthy volunteers. In the future, applying tACS over the bilateral M1 in the stomatognathic system may aid in the treatment of OMD given the bilateral cortical control of this system. Indeed, in our previous study, tDCS of the bilateral M1 area representing the tongue region significantly increased the excitability of this region and improved tongue motor functions compared to the application of tDCS to the unilateral M1 of the tongue region in healthy volunteers [123].

## 6. Conclusions

In this review, we provide an overview of the unique central spatiotemporal processing mechanisms in the stomatognathic system. The uncertain effects of DBS on OMD may, in part, be explained by the unique characteristics of the bilateral central regulation of sensorimotor functions in the stomatognathic system. Further exploration of the neurophysiological underpinnings of stomatognathic functions will lead to an improved understanding of the etiology of OMD and may lead to innovative approaches for future symptomatic and disease-modifying treatments.

## 7. Methods

This literature review was conducted based on the comprehensive analysis of electronic medical literature databases (PubMed, Scopus, EMBASE, and Google scholar) prior to 1 September 2022. Search keywords included oromandibular dystonia, orofacial dystonia, mandibular dystonia, jaw dystonia, lingual dystonia, Meige syndrome, botulinum toxin, botulinum toxin therapy, deep brain stimulation, transcranial direct current stimulation (tDCS), and transcranial alternating-current stimulation (tACS) for Section 1, Section 4 and Section 5 of the manuscript. We also searched the terms trigeminal nerve, lingual nerve, tongue, palate, face, lip, oromandibular, stomatognathic system, sensory disturbance, sensory abnormality, electroencephalography (EEG), and magnetoencephalography (MEG) for Section 1, Section 2 and Section 3 and Section 5. Each search result was independently reviewed for eligibility by the author (H.M.). No restriction was placed with respect to the original text language.

## Data Availability

Not applicable.
